# Noise Assessment in Intensive Care Units

**DOI:** 10.1016/S1808-8694(15)30547-4

**Published:** 2015-10-19

**Authors:** Ivan Senis Cardoso Macedo, Daniela Cunha Mateus, Eduardo De Martin Guedes C Costa, Ana Cristina Lanfranchi Asprino, Edmir Américo Lourenço

**Affiliations:** 1 Physician, trained at the Jundiai Medical School - Sao Paulo; 2 4th year medical student at the Jundiai Medical School - Sao Paulo; 3 4th year medical student at the Jundiai Medical School - Sao Paulo; 4 1st year medical resident in Otorhinolaryngology, Jundiai Medical School - Sao Paulo; 5 Department of Otolaryngology, Jundiai Medical School - Sao Paulo. Jundiai Medical School, Santo André - Sao Paulo

**Keywords:** environmental statistics, noise, intensive care units

## Abstract

Intensive Care Units are environments with numerous noise sources. In different hospital environments it is recommended to have a sound pressure level between 35 and 45db(A).

**Aim:**

To measure the sound pressure levels in three ICU at a hospital in Jundiaí, State of São Paulo, Brazil.

**Study Design:**

Observational.

**Materials and Methods:**

we used a Minipa model MSL1532C (USA) sound meter, according to the Brazilian Technical Standards (NBR 10151), in order to measure sound levels in the ICUs at different moments, that is, the morning, afternoon and night at peak times of activity.

**Results:**

The values found during the checking of the sound pressure levels were 64.1dB (A) in the First ICU, 58.9 dB (A) in the Coronary Unit and 64dB (A) in the second ICU.

**Conclusion:**

High sound pressure levels in ICU still mean an important health-related problem for patients in these units. None of the three ICU pad levels above 85dB, showing that there is no occupational risk for the health care teams in the environments studied.

## INTRODUCTION

Intensive care units (ICUs) contain many sources of noise, such as aspirators, monitors, mechanical ventilators, computers, printers, air conditioning vents, and others. Elevated sound pressure levels may cause stress, psychological disorders, and sleep disorders.^1^ Psychological effects due to loud noise may cause behavioral disorders and physiological responses to stress. Noise may also alter assessments of sedation.^2^, ^3^ The hypothalamicpituitary-adrenal system is sensitive to a sound pressure level of 65 dBA; at this level, high amounts of adrenalin, noradrenalin and corticosteroids are secreted, blood pressure may be elevated, the heart rate may be altered and there may be peripheral vasoconstriction.^3^ A further important issue is synergism between ototoxic drugs and high sound pressure levels, which increase the risk of hearing loss in patients.^4^, ^5^, ^6^, ^7^ Comfortable noise levels are underestimated in hospitals; finding solutions to improve environmental quality - as related to the level of noise -when designing hospitals has been markedly difficult. The United States Environmental Protection Agency and the Brazilian Technical Standards Association (ABNT) have recommended sound pressure levels ranging from 35 to 45db (A) in various hospital settings.^8^, ^9^

The aim of this study was to measure sound pressure levels in three intensive care units (ICUs) of a Teaching Hospital in Jundiai, Sao Paulo State, Brazil.

## METHODS

A Minipa model MSL-1532C (USA) model was used to measure sound levels in a first ICU, a second ICU, and a Coronary Care Unit (CCU) located in a hospital in Jundiai, Sao Paulo state, according to ABNT standards (NBR 10151). Data gathering and analysis were done following an evaluation and consent of the ICU directors and approval by the Institutional Review Board (protocol approval number FR-226570). Only the physician in charge in each shift was aware of the strategic placement of the sound level measuring device. Measurements were done during two hours per session (morning, afternoon and evening); the number of staff members in the environment being studied was recounted every 5 minutes. Data were analyzed with descriptive statistics using Microsoft's® Excel 2000 software.

## RESULTS

Measurement sound pressure level values were 64.1 dB (A) in the first ICU, 58.9 dB (A) in the CCU and 64 dB (A) in the second ICU. Table 1 shows minimum and maximum sound pressure levels, the mean number of staff on site, and the number of patients in each ICU.

**Table 1:** Description of parameters analyzed according to each unit.

Parameter	Site		
	First ICU	CCU	Second ICU
Mean - dB (A)	64.1	58.9	64.0
Low - dB (A)	57.0	51.9	55.9
High - dB (A)	80.4	73.3	82.4
Standard deviation	3.4	2.9	3.1
Mean staff number on site	13	8	14
Number of patients	8	7	13

CCU: Coronary Care Unit, ICU: Intensive Care Unit.

Table 2 shows sound pressure levels as percentages above recommended values; measured according to the site.

**Table 2:** Percentage above recommended sound pressure levels measured in each ICU.

Parameter	Local		
	First ICU	CCU	Second ICU
Mean - dB (A)	42.4%	30.8%	42.2%
Minimum	26.7%	15.4%	24.2%
Maximum	78.7%	62.8%	83.1%

CCU: Coronary Care Unit, ICU: Intensive Care Unit.

## DISCUSSION

All ICUs in this study had mean sound pressure levels at least 30% over recommended values (ABNT and United States Environmental Protection Agency).^8^, ^9^ at no moment during measurements were these values within normal parameters for such institutions; this is a contributing factor to an increased morbidity in patients. Elevated sound pressure levels in hospital environments is common worldwide, as several studies have reported: a mean 60 to 65 dB (A) in an Austrian hospital, 55 dB (A) in the University of Valencia hospital, Spain, and 68 dB (A) in a hospital ICU in Manitoba, Canada;1 these findings are similar to our results.

Noise generated in ICUs has increased in recent decades due to a substantial increase in the number of devices with acoustic alarms, and background noise by actions and speaking of healthcare professionals. Patients and healthcare staff may be affected alike.^3^ There is a high risk of chronic occupational noise exposure, especially with complex tasks. Excessive and continuous noise exceeding 85 dB may cause physiological and psychological harm in healthcare teams, such as hypertension, altered heart rates and muscle tone, headaches, hearing loss, mental confusion, low attention span and irritability.^3^ However, we measured no values above 85dB in this study, showing that there was no occupational health risk for staff in the environments that were investigated.

The design of devices for intensive care units should take noise reduction into account. Noise should be a concern in procurement, installation and use of such devices, as changes at a later date may increase costs.^10^ Thus, awareness of this issue is important for critical care professionals and manufacturers of medical equipment. The amount of noise generated by each device should be established for efficient noise-reducing measure to be adopted, as noise in a workplace may originate from several sources.^1^, ^3^ Guidelines are important, as evidence suggests their relevance as part of training programs for healthcare professionals when attempting to change work sites.^4^ The main issue in such programs is controlling excessive noise to reduce its effects on human hearing, to decrease stress, and to have positive effects on healing. Information for healthcare team helps trigger behavioral changes. Important measures in routine work are: avoiding loud conversations close to patients, generating specific environments for clinical discussions; controlling bells, alarms, cell phones, pagers, televisions and radios, use of posters in critical areas, defining silence periods for sleep hours, during which sound alarms should be turned down or off if possible, and nursing interventions should be kept to a minimum in clinically stable patients. Other measures include collaboration of supporting staff (e.g.: janitors, laboratory and radiology technicians), and implementing continued education programs for ICU staff. Environmental measures include adjusting the ICU architecture by adding noise-absorbing floors, ceilings, and walls, physical separators between beds in larger units, rubber seals on doors and windows, and noise assessments prior to acquiring equipment.^3^

## CONCLUSION

Sound pressure levels above ABNT and United States Environmental Protection Agency recommended values were found in three intensive care at a hospital in Jundiai, Sao Paulo State, Brazil, emphasizing a major inpatient morbidity issue.

At no time did the three ICUs have sound pressure levels over 85 dB, suggesting no occupational health risk to healthcare professionals at those sites.
